# Genome survey and high-resolution backcross genetic linkage map construction of the ridgetail white prawn *Exopalaemon carinicauda* applications to QTL mapping of growth traits

**DOI:** 10.1186/s12864-019-5981-x

**Published:** 2019-07-22

**Authors:** Jitao Li, Jianjian Lv, Ping Liu, Ping Chen, Jiajia Wang, Jian Li

**Affiliations:** 1 Ministry of Agriculture and Rural Affairs, Yellow Sea Fisheries Research Institute, CAFS, Key Lab for Sustainable Development of Marine Fisheries, Qingdao, 266071 China; 2 Function Laboratory for Marine Fisheries Science and Food Production Processes, National Lab for Ocean Science and Technology, Qingdao, 266071 China

**Keywords:** *Exopalaemon carinicauda*, Genome, SNP, High-resolution linkage map, QTL mapping

## Abstract

**Background:**

High-resolution genetic linkage map is critical for QTL mapping, genome sequence assembly and marker-assisted selection in aquaculture species. The ridgetail white prawn *Exopalaemon carinicauda* is one of the most economic shrimp species naturally distributed in the coasts of eastern China and western Korea. However, quite limited genomics and genetics information have been exploited for genetic improvement of economic traits in this species.

**Results:**

In the present study, we conducted genome survey and constructed high-resolution genetic linkage maps of the ridgetail white prawn with reciprocal-cross mapping family genotyped using next-generation sequencing approaches. The estimated genome size was 9.33 Gb with a heterozygosity of 0.26% and a repeat sequence ratio of 76.62%. 65,772 protein-coding genes were identified by genome annotation. A total of 10,384 SNPs were used to high-throughput genotyping and assigned to 45 linkage groups (LGs) from reciprocal backcross families of *E. carinicauda*, and the average marker distances were 0.73 cM and 0.55 cM, respectively. Based on the high-resolution linkage map, twenty-three QTLs related to five growth traits were detected. All QTLs could explain 8.8–15.7% of the total growth-traits variation.

**Conclusions:**

The genome size of *E. carinicauda* was estimated more accurately by genome survey analysis, which revealed basic genomic architecture. The first high-resolution backcross genetic linkage map and QTLs related to growth traits will provide important information for QTL fine mapping, genome assembly and genetic improvement of *E. carinicauda* and other palaemon shrimps.

**Electronic supplementary material:**

The online version of this article (10.1186/s12864-019-5981-x) contains supplementary material, which is available to authorized users.

## Background

The ridgetail white prawn *Exopalaemon carinicauda* is an important economic shrimp species naturally distributed in the coasts of eastern China and western Korea [1]. Due to multiple advantages of fast growth, good reproductive performance and strong adaptability to the environment, the aquaculture scale of the ridgetail white prawn expanded rapidly and contributed to one third of the total output of the polyculture ponds in eastern China [2]. The annual aquaculture area and yield of *E. carinicauda* in China are about 20, 000 ha and 45, 000 tons, respectively. But the aquaculture of *E. carinicauda* mainly relyed on natural seeds and wild spawning broodstock, which resulted in unclear genetic background and germplasm degradation, seriously affecting industrial development. Conventional breeding methods based on selection of individuals on phenotypic values. The approaches of using molecular markers for selection, or by both markers and other phenotypic data, named marker-assisted selection (MAS) [3] was first applied when the restriction fragment length polymorphisms (RFLP) were detected in economic species [4]. MAS is especially helpful for traits that are difficult to measure, exhibit low heritability and/or are expressed late in development process. Implementation of MAS requires DNA markers that are tightly linked to quantitative trait loci (QTL) for economic traits based on genetic linkage maps [5]. Therefore, MAS approaches is urgently required for sustainable development in culture of *E. carinicauda*.

High-resolution genetic linkage map is necessary for genome assembly, as well as for mapping QTL of economic traits [6]. In the past decade, genetic linkage map had been constructed using various molecular markers in multiple aquaculture species [5, 7–10], including Tilapia, Rainbow trout, Atlantic salmon, Asian seabass, Half tongue sole, Channel catfish, Common carp, Japanese flounder and many others. Linkage map provide essential tools for QTL localization and promote MAS process in multiple aquaculture species. For example, growth traits have been mapped in Rainbow trout [11], Asian seabass [12], Salmons [13]; *Vibrio anguillarum* and lymphocystis disease-resistant traits in Japanese flounder have been successfully mapped and applied to marker-assistant breeding [9, 14]; sex-determination traits have been localized by QTL mapping approaches in Half tongue sole [7], Tilapia [15] and Atlantic halibut [16]. Recently, High-resolution genetic linkage maps have been constructed in crustaceans, such as Kuruma prawn [17], Chinese shrimp [18], Black tiger shrimp [19], Pacific white shrimp [20], Swimming crab [21] and Chinese mitten crab [22]. In contrast, a high-resolution linkage map is urgently needed for genomic and genetic researches in the ridgetail white prawn.

Next-Generation Sequencing (NGS) technology and associated genotyping advancements have become widely used to implement de novo genome sequencing and high-resolution linkage map construction in non-model species. In recent years, Specific-Length Amplified Fragment sequencing (SLAF-seq) has been successfully applied in large-scale de novo SNP discovery and genotyping in various species [23]. As a reliable, high throughput and double-barcode genotyping platform, SLAF-seq is fast, accurate and cost-effective for high-resolution linkage maps construction, such as sesame [24], soybean [25, 26] and cucumber [27]. For aquaculture species, a high-resolution linkage map, including 5,885 markers, was constructed in common carp using SLAF-seq with marker intervals of 0.68 cM on average [23]. High-resolution genetic map of the Pacific white shrimp was also constructed using this method with an average marker distance of 0.7 cM [20]. The main purposes of constructing high-resolution linkage map is to conduct the mapping of QTLs related to economic traits. Based on the high-resolution linkage map, several QTLs related to body length and body weight of shrimps were detected [20, 22].

Constructing a genetic linkage map requires reference populations/families where molecular markers segregate [28]. Because common crustaceans reproduced one generation per year, the most of reference populations of shrimps and crabs were F_1_ and F_2_ families [17–22]. Backcross populations, derived by crossing the F_1_ hybrid to one of their parents, are usually used to construct genetic linkage map and QTL mapping due to direct reflection of separation of F_1_ gametes and higher mapping efficiency than F_2_ population [11, 29]. In the present study, we constructed reciprocal-cross backcross population of *E. carinicauda* for the first time based on the characteristics of multiple reproduction annually. We then conducted genome survey analysis and construction of a high-resolution linkage map to investigate the genomic and genetic architecture of *E. carinicauda*. Based on the high-resolution linkage map, QTL mapping was also conducted to detect markers related to growth traits.

## Results

### Genome survey of *E. carinicauda*

**Fig. 1:**
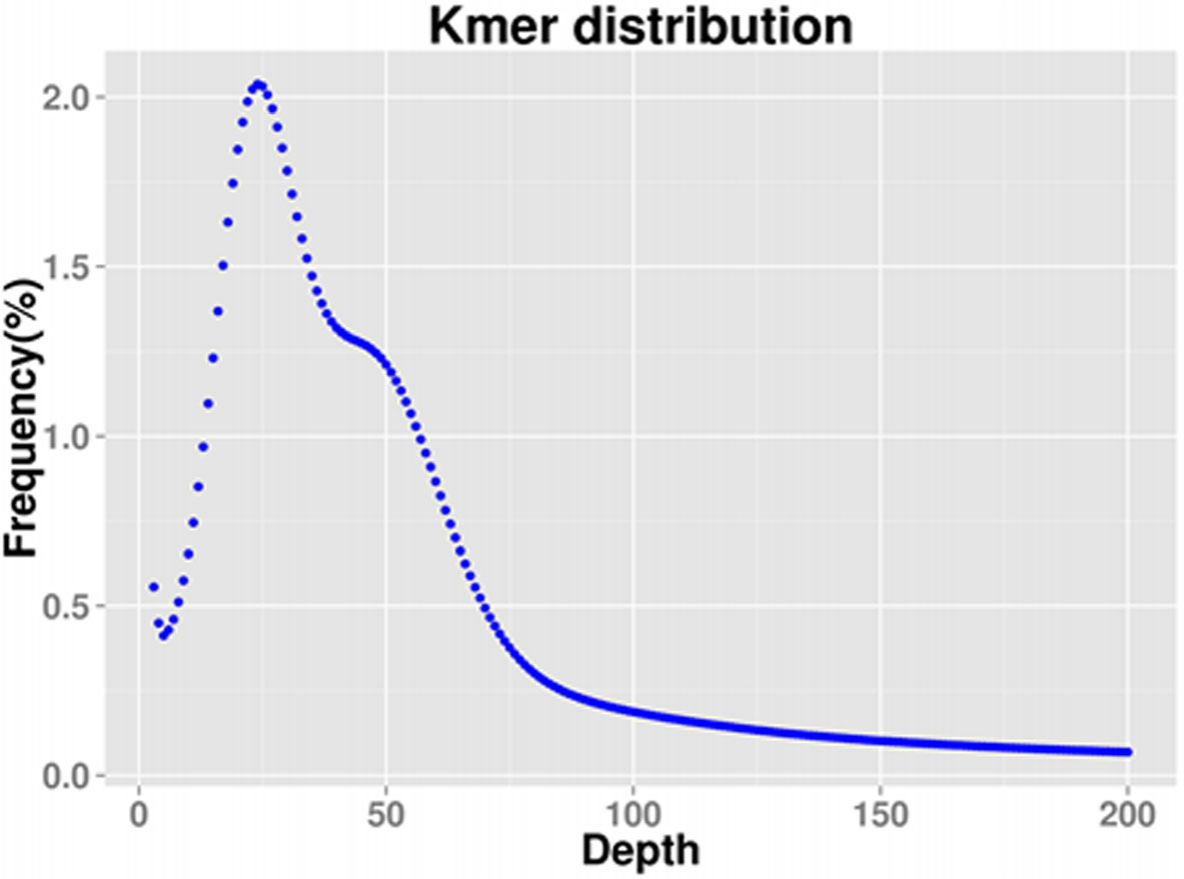
Kmer distribution of *E. carinicauda* genome

We constructed six 270 bp DNA libraries with short paired-end and sequenced them by Illumina Hiseq 4000 platform. 294.46 Gb high-quality reads with 40.01% GC content were obtained in total after sequencing, which covered approximately 31.57-fold the genome size of *E. carinicauda*. Sequencing raw data have been submitted to the Nucleotide database of NCBI with the accession number QUOF000000000.1. K-mer curve was obtained based on the frequencies of 23-mers (nucleotide strings with a length of 23 bp) within sequencing data (Fig. 1). K-mer analysis revealed that there was a peak at the K-mer depth of 24. The genome size of *E. carinicauda* was estimated as 9.33 Gb with remarkably low heterozygosity (0.26%), which were possessed by approximately 76.62% repeat sequences (Table 1). The total length of assembled scaffolds was 9.18 Gb, which covered approximately 98.40% of *E. carinicauda* genome (Table 2). Finally, we predicted 65,772 genes (Additional file 2: Table S2) based on sequencing contigs/scaffold.

**Table 1:** Statistics of the genome survey from *E. carinicauda*

*Items*	
Genome size (Gb)	9.33
Heterozygosity	0.26%
Repeat sequences	76.62%
GC content	40.01%
Data (Gb)	294.46
Depth (X)	31.57

**Table 2:** Statistics of the genome assembly from *E. carinicauda*

*Items*	*Contig*	*Scaffold*
Number	31,371,476	28,089,718
Total Length (bp)	9,085,107,780	9,185,133,106
100–300	6,721,269,034	6,821,205,337
300–500	1,196,072,737	1,002,403,357
500–1000	793,254,189	809,021,501
1000–2000	297,655,772	395,241,325
> 2000	76,855,759	157,261,587
Longest	135,963	135,963
Shortest	100	100
Mean Length (bp)	290	327
N50 (bp)	422	586
N90 (bp)	115	116

### Features of the phenotypic traits

The mean values of body length (*BL*), body weight (*BW*), carpace length (*CL*), carpace width (*CW*) and carpace height (*CH*) in backcross family were 39.25 ± 5.78 mm, 0.91 ± 0.41 g, 20.42 ± 3.57 mm, 5.54 ± 0.82 mm and 6.27 ± 0.94 mm, respectively (Additional file 1: Table S1). And the average values of body length (*BL*), body weight (*BW*), carpace length (*CL*), carpace width (*CW*) and carpace height (*CH*) in reverse backcross family were 34.79 ± 3.97 mm, 0.62 ± 0.22 g, 18.20 ± 2.56 mm, 4.88 ± 0.62 mm and 5.56 ± 0.60 mm, respectively. The growth-related traits showed strong correlations with each other (r = 0.774–0.983, *P* < 0.001 for all) (Tables 3 and 4). The highest correlation value (r = 0.983) was existed between *BL* and *BW*. The *BW* strongly correlated with *BL* (r = 0.983), *CH* (r = 0.970) and *CW* (r = 0.962).

**Table 3:** Pearson correlation coefficients (r) for all pairwise combinations of the five growth-related traits (*P* < 0.001 for all) in B backcross family

*Traits*	*BL*	*CL*	*CW*	*CH*	*BW*
*BL*	1				
*CL*	0.946	1			
*CW*	0.967	0.911	1		
*CH*	0.967	0.915	0.970	1	
*BW*	0.975	0.924	0.962	0.970	1

Note: *BL* body length, *CL* carpace length, *CW* carpace width, *CH* carpace height, *BW* body weight, the same below

**Table 4:** Pearson correlation coefficients (r) for all pairwise combinations of the five growth-related traits (*P* < 0.001 for all) in B′ reverse backcross family

*Traits*	*BL*	*CL*	*CW*	*CH*	*BW*
*BL*	1				
*CL*	0.919	1			
*CW*	0.837	0.774	1		
*CH*	0.934	0.913	0.816	1	
*BW*	0.983	0.920	0.823	0.925	1

### SLAF-seq library construction and sequencing

A total of 204 SLAF-seq libraries from 4 parents and 200 backcross offsprings were constructed and sequenced on 4 lanes of Illumina HiSeq 2500 platform to generate 4.98 billion raw reads. After data trimming, 100.60 Gb of sequencing data, were individually divided into SLAF tags according to their MIDs. Finally, female and male parental data sets in cross BC_1_ family, containing respectively 11.26 million filtered reads (comprising 2.25 Gb of data with a GC% of 42.74) and 7.40 million filtered reads (comprising 1.48 Gb of data with a GC% of 43.98), were correspondingly partitioned into 273667 and 248178 SLAF tags. And female and male parental data sets in reverse cross BC_1_ family were correspondingly partitioned into 277696 and 251322 SLAF tags. From the 100 offspring of BC_1_ family, a total of 2.39 billion reads (with an average GC% of 42.09) corresponding to 482,281.16 Mb of data. For the 100 offspring of reverse cross BC_1_ family, a total of 2.21 billion reads (with an average GC% of 42.30) corresponding to 449238.48 Mb of data.

### SNP marker genotyping and genetic linkage map

**Fig. 2:**
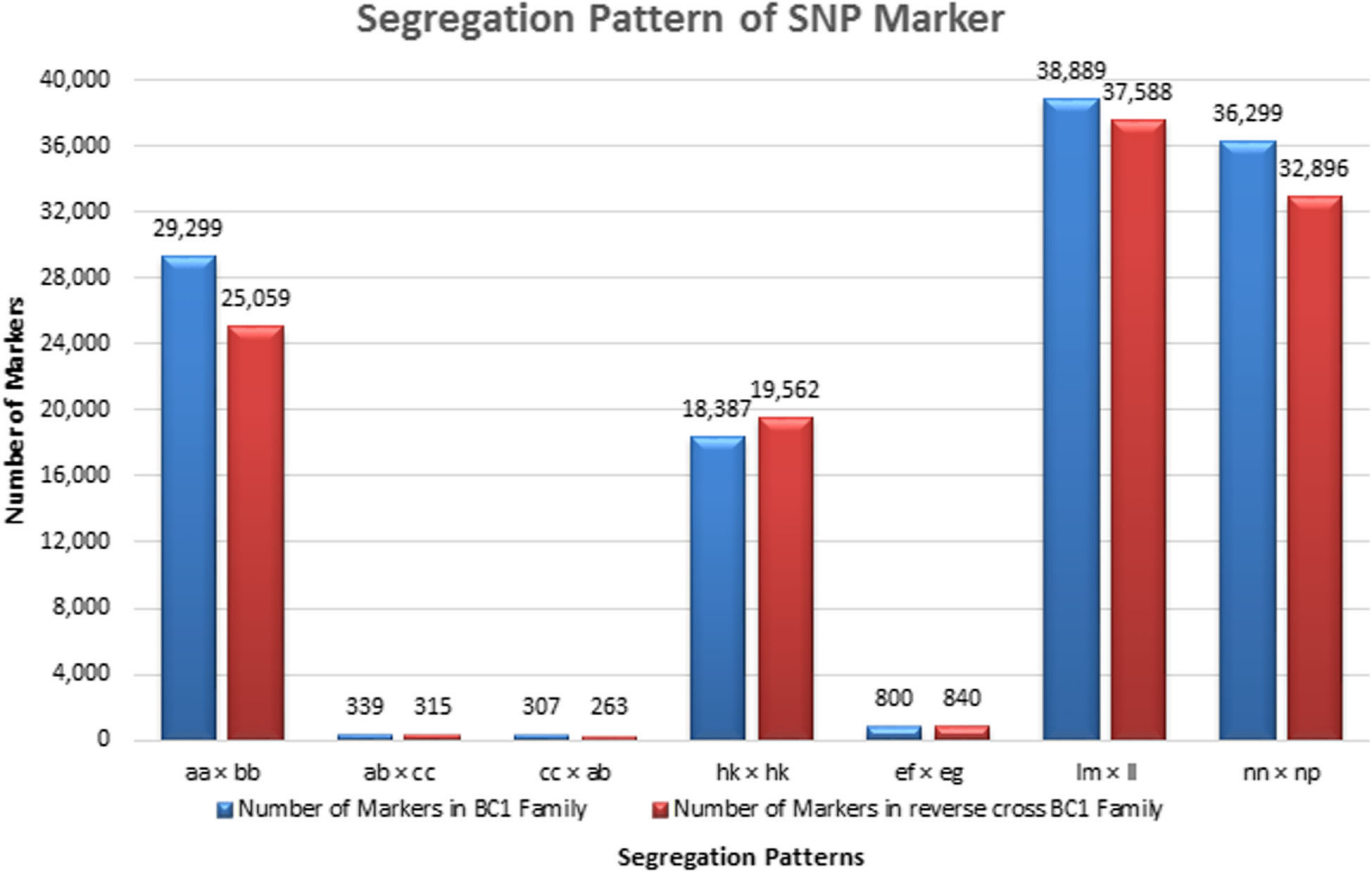
Segregation pattern of SNP markers from *E. carinicauda*

After SLAF sequencing, 497888010 paired-end reads were generated for the mapping family (four parents and 200 progenies). A total of 301,314 polymorphic SLAF markers were identified from 1433342 SLAF markers, of which 10384 SNP markers could be successfully genotyped in both parents and offspring (Table 5). SNP markers with the seven segregation patterns (aa × bb, ab × cc, cc × ab, hk × hk, ef × eg, lm × ll, nn × np) could be used in linkage map construction for the BC_1_ family, of which aa × bb was one of the major pattern and most efficient markers for map construction (Fig. 2). The average read depth in backcross family of genotyped markers were 6.52, 15.36 and 22.75 in the offspring, male and female parents, respectively (Additional file 3: Table S3). And the average read depth in reverse backcross family of genotyped markers were 7.09, 16.90 and 28.51 in the offspring, male and female parents, respectively (Additional file 4: Table S4).

**Table 5:** Statistics of SLAF and SNP data

Items	Backcross family	Reverse Backcross family
Number of reads	257,646,490	240,241,520
Number of SLAF	835,181	598,161
Polymorphic SLAF	164,444	136,870
Polymorphic SNP	4050	6334
Average depth in male parent	15.36	16.9
Average depth in female parent	22.75	28.51
Average depth in offspring	6.52	7.09

**Fig. 3:**
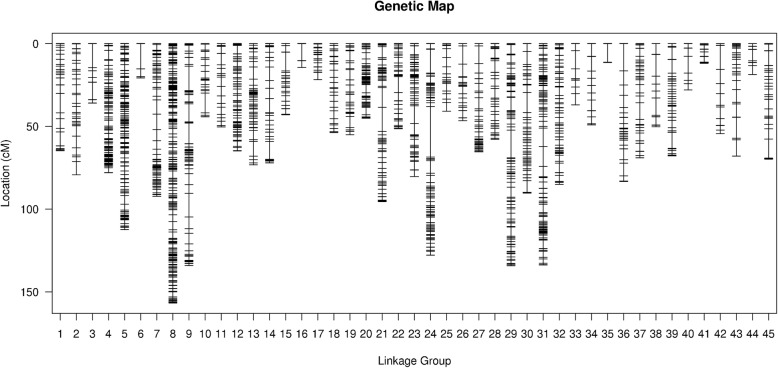
The genetic linkage map of BC_1_ family in *E. carinicauda*

**Fig. 4:**
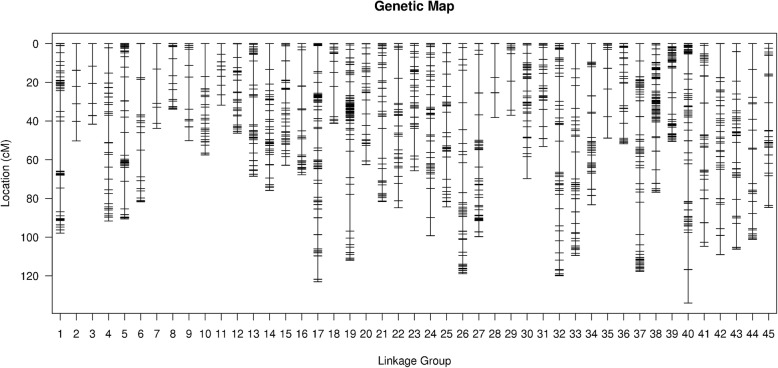
The genetic linkage map of reverse BC_1_ family in *E. carinicauda*

High-resolution SLAF-based SNP genetic maps of *E. carinicauda* based on backcross populations were first constructed using the pseudo-testcross strategy. The BC_1_ family genetic linkage map contained 45 linkage groups with 4050 markers and the reverse cross BC_1_ family genetic linkage map contained 45 linkage groups with 6,334 markers (Table 6, Fig. 3, Fig. 4, Additional file 5: Table S5 and Additional file 6: Table S6). The total map distance of two maps were 2939.27 cM with an average inter-locus distance of 0.73 cM and 3460.28 cM with an average inter-locus distance of 0.55 cM respectively, which covered 97.65 and 98.44% respectively of the genome based on the total length of the genome.

**Table 6:** Characteristics of genetic maps of *E. carinicauda* Backcross families

LG_ID	Map of Backcross family			Map of Reverse Backcross family		
	Total marker	Total Distance(cM)	Average Distance(cM)	Total marker	Total Distance(cM)	Average Distance(cM)
1	61	64.70	1.08	134	97.93	0.74
2	65	79.28	1.24	61	50.22	0.84
3	49	35.89	0.75	64	41.62	0.66
4	152	78.00	0.52	147	91.59	0.63
5	203	112.36	0.56	326	90.52	0.28
6	36	20.73	0.59	92	81.75	0.90
7	149	92.27	0.62	71	43.80	0.63
8	263	156.72	0.60	128	33.88	0.27
9	159	134.13	0.85	66	50.11	0.77
10	65	44.18	0.69	62	57.45	0.94
11	78	50.37	0.65	66	31.75	0.49
12	116	64.73	0.56	99	46.28	0.47
13	61	73.24	1.22	156	68.49	0.44
14	80	72.07	0.91	125	75.86	0.61
15	65	42.98	0.67	120	62.85	0.53
16	42	14.39	0.35	103	67.73	0.66
17	34	21.80	0.66	326	123.02	0.38
18	34	53.71	1.63	89	41.19	0.47
19	56	55.01	1.00	199	111.98	0.57
20	111	45.13	0.41	79	62.46	0.80
21	124	95.42	0.78	129	81.59	0.64
22	86	51.35	0.60	86	84.83	1.00
23	123	80.31	0.66	128	65.67	0.52
24	156	127.80	0.82	133	99.18	0.75
25	39	40.86	1.08	159	84.33	0.53
26	55	46.57	0.86	175	118.87	0.68
27	201	65.40	0.33	183	99.70	0.55
28	105	57.82	0.56	66	38.16	0.59
29	145	134.27	0.93	73	37.02	0.51
30	106	90.32	0.86	166	69.72	0.42
31	200	133.71	0.67	125	53.11	0.43
32	108	85.05	0.79	259	119.91	0.46
33	38	37.02	1.00	98	109.44	1.13
34	50	49.18	1.00	166	83.23	0.50
35	35	11.40	0.34	81	48.79	0.61
36	90	83.25	0.94	101	51.73	0.52
37	68	69.00	1.03	206	117.64	0.57
38	54	50.17	0.95	106	76.79	0.73
39	84	67.86	0.82	193	50.52	0.26
40	38	28.04	0.76	437	133.99	0.31
41	32	11.85	0.38	124	104.72	0.85
42	63	54.44	0.88	163	108.97	0.67
43	50	67.97	1.39	211	106.13	0.51
44	32	18.70	0.60	176	101.18	0.58
45	89	69.82	0.79	77	84.58	1.11
Total	4,050	2,939.27	0.73	6,334	3,460.28	0.55
Average	90	65.32	0.73	140.75	76.90	0.55
Genome coverage (%)		97.65%			98.44%

### Genetic linkage map integration

**Fig. 5:**
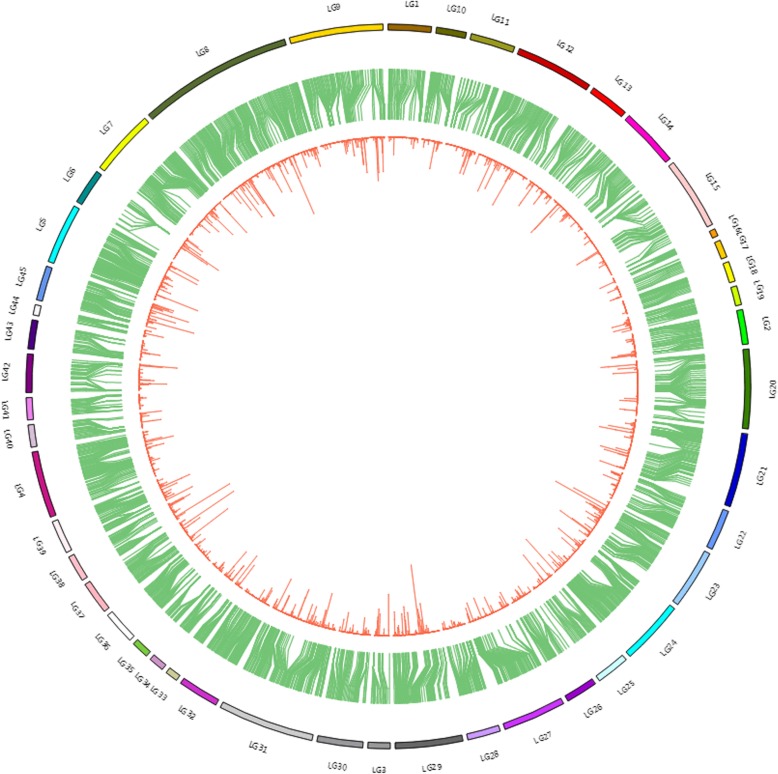
The integrated map of genome, BC_1_ map and transcriptome in *E. carinicauda.* Outer ring, the linkage group; Intermediate ring, contigs or scaffolds of genome assembly aligned with markers from the linkage map; Inner ring, unigene sequences of transcriptome aligned with scaffold/contig sequences

**Fig. 6:**
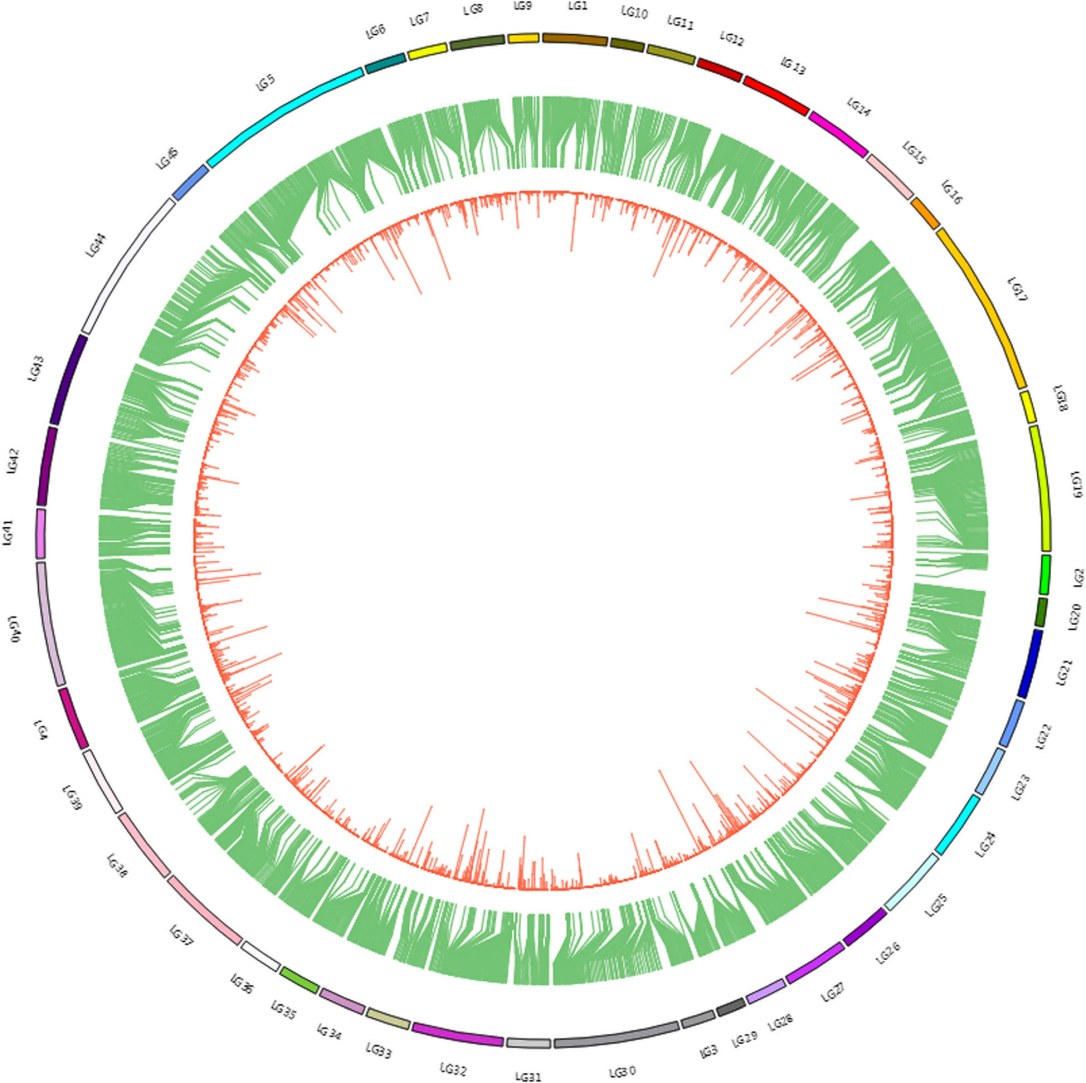
The integrated map of genome, reverse BC_1_ map and transcriptome in *E. carinicauda.* Outer ring, the linkage group; Intermediate ring, contigs or scaffolds of genome assembly aligned with markers from the linkage map; Inner ring, unigene sequences of transcriptome aligned with scaffold/contig sequences

Among the 4050 markers in the backcross map, 2733 markers could align to 1468 genomic scaffolds/contigs with high confidence (Additional file 7: Table S7). And 91 markers were anchored to unigenes of transcriptome (Additional file 8: Table S8). In the reverse backcross map, 4,297 markers could align to 2403 genomic scaffolds/contigs with high confidence (Additional file 9: Table S9). And 156 markers were anchored to unigenes of transcriptome (Additional file 10: Table S10). Based on blast information, there were some markers of genetic linkage maps, genomic scaffolds/contigs and unigenes of transcriptome that can be integrated together (Fig. 5, Fig. 6). 7,030 markers could be aligned to the genomic scaffolds/contigs or transcriptome unigenes, of which 846 markers could be blasted via with the public databases.

### QTL mapping of growth-related traits

**Fig. 7:**
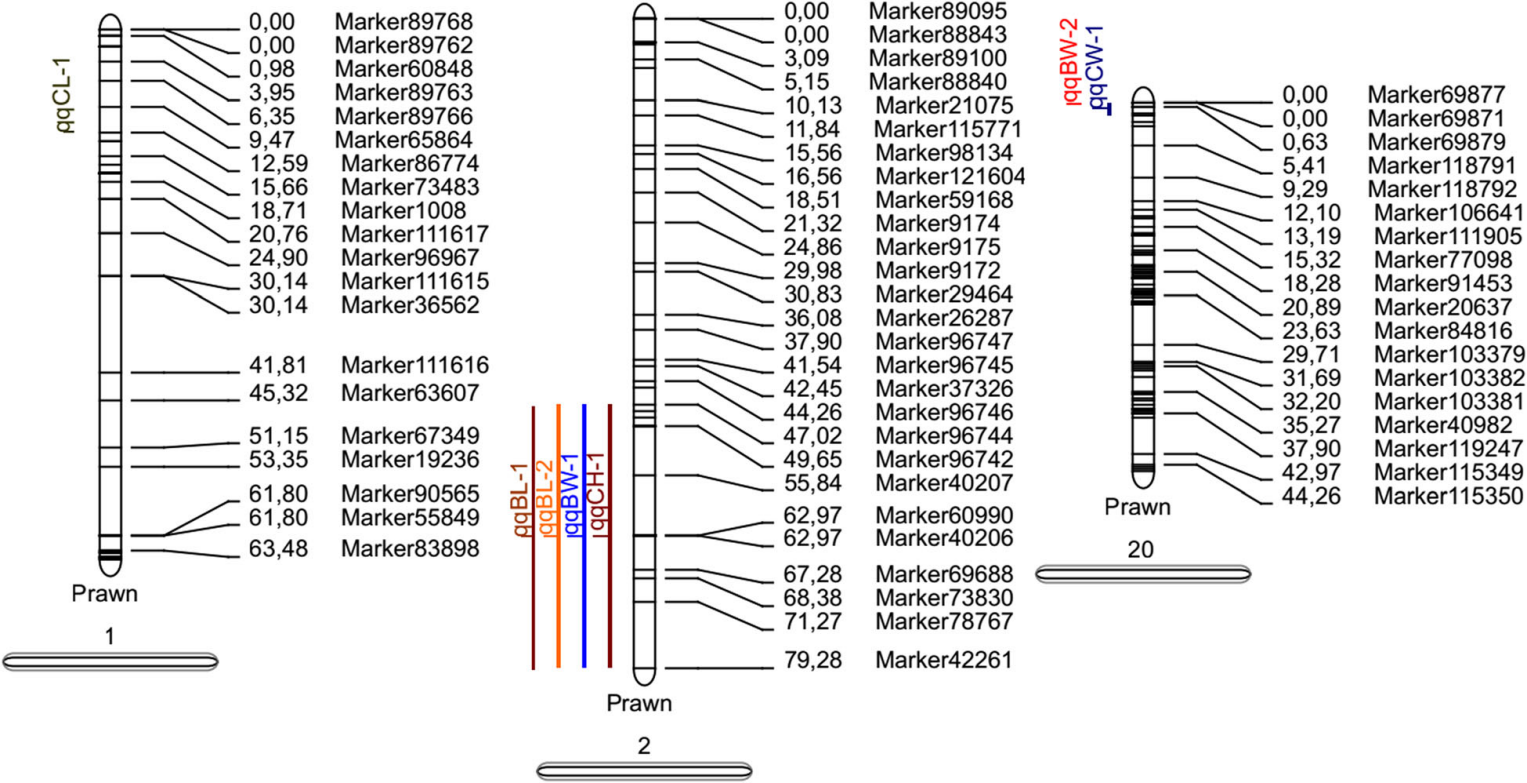
The QTLs related to growth traits in the BC_1_ map of *E. carinicauda.* QTLs of different growth traits is represented by different colors

**Fig. 8:**
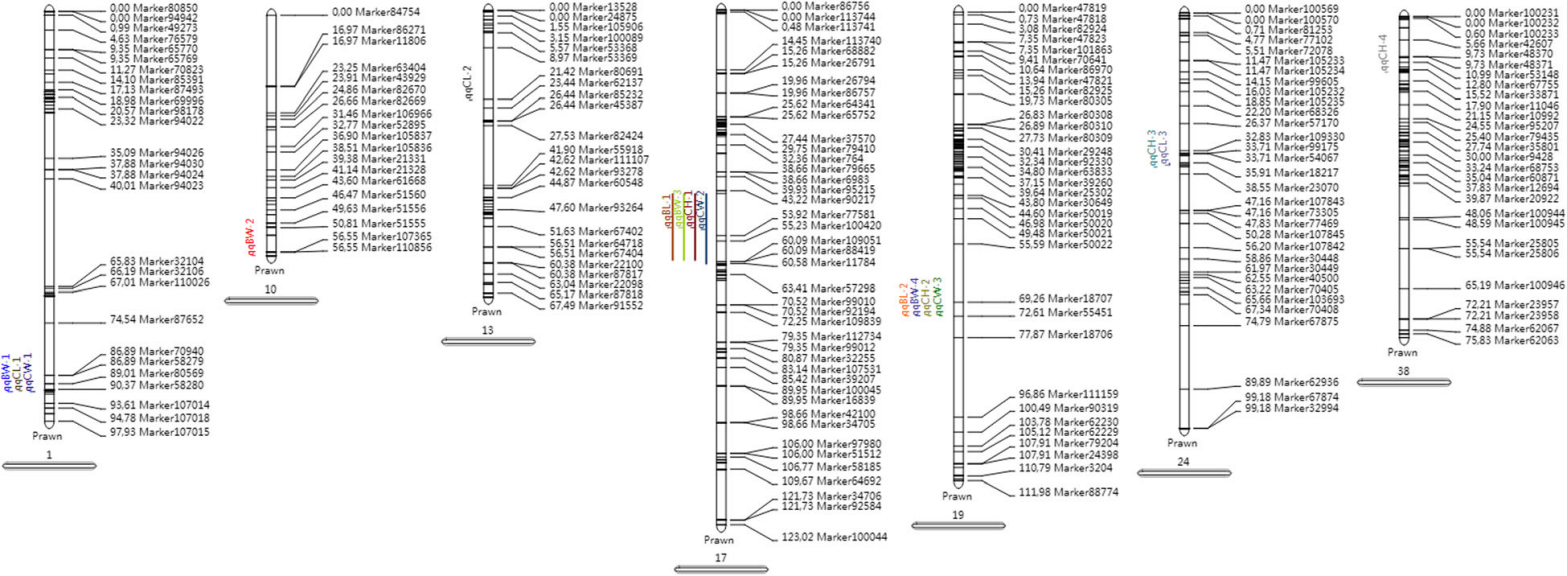
The QTLs related to growth traits in the reverse BC_1_ map of *E. carinicauda.* QTLs of different growth traits is represented by different colors

The high-resolution genetic linkage map was used in the present study for QTL mapping of growth-related traits using MapQTL 4.0 software. In the map of Backcross family, 7 QTLs were detected for all the five growth-related traits, which were distributed on LG1, LG2 and LG20 (Table 7 and Fig. 7). These QTLs with LOD values of 2.66–3.62 contributed to *PVE* of 11.5–15.7%. Among them, *qBW-1* located at 49.652–79.283 cM of LG2 with the highest LOD score of 3.62, and correspondingly had the higher *PVE* value of 15.4%. Some QTL intervals were clustered together on the same respective linkage groups (LGs). One major cluster containing three QTLs (*qBL-2, qCH-1* and *qBW-1*) was detected between the positions of 47.024–79.283 cM on LG2. On LG20, another cluster located within the region (0–1.826 cM) also consisted of two QTLs (*qCW-1* and *qBW-2*). A total of 16 markers were located in the QTL intervals, among which, 8 markers (50%) distributed on LG2. In the map of reverse Backcross family, a total of 16 QTLs were detected for all the five growth-related traits, which were distributed on LG1, LG10, LG13, LG17, LG19, LG24 and LG38 (Table 7 and Fig. 8). The QTLs with LOD values of 2.0–2.74 contributed to *PVE* of 8.8–11.9%. Of which, one major cluster containing four QTLs (*qBL-2, qCW-3, qCH-2* and *qBW-4*) was detected at the positions of 72.613 cM on LG19. Another cluster situated within the region (43.872–60.091 cM) on LG19 also consisted of four QTLs (*qBL-1, qCW-2, qCH-1* and *qBW-3*). A total of 25 markers were located in the QTL intervals, among which, 10 markers (40.0%) distributed on LG10.

**Table 7:** Characteristics of growth related QTLs

Maps	Traits	QTL	Linkage Group	Start (cM)	End (cM)	Marker Number	Max LOD	Max PVE
*Backcross map*	*BL*	*qBL-1*	2	47.024	47.024	1	3.09	13.3
		*qBL-2*	2	79.283	79.283	1	3.00	12.9
	*CL*	*qCL-1*	1	12.586	12.586	1	2.66	11.5
	*CW*	*qCW-1*	20	0	1.826	7	3.57	15.7
	*CH*	*qCH-1*	2	47.024	79.283	3	3.27	14.0
	*BW*	*qBW-1*	2	49.652	79.283	8	3.62	15.4
		*qBW-2*	20	0	0	2	3.26	14.3
*Reverse backcross map*	*BL*	*qBL-1*	17	43.872	60.091	2	2.36	10.3
		*qBL-2*	19	72.613	72.613	1	2.74	11.9
	*CL*	*qCL-1*	1	90.716	90.716	1	2.12	9.3
		*qCL-2*	13	21.418	21.418	1	2.04	9.0
		*qCL-3*	24	35.908	36.779	4	2.24	9.8
	*CW*	*qCW-1*	1	90.716	91.096	2	2.12	9.3
		*qCW-2*	17	43.872	60.995	7	2.38	10.4
		*qCW-3*	19	72.613	72.613	1	2.0	8.8
	*CH*	*qCH-1*	17	43.872	60.091	2	2.15	9.4
		*qCH-2*	19	72.613	72.613	1	2.26	9.9
		*qCH-3*	24	36.779	36.779	2	2.05	9.0
		*qCH-4*	38	13.293	13.293	1	2.14	9.4
	*BW*	*qBW-1*	1	90.716	91.096	2	2.09	9.2
		*qBW-2*	10	57.446	57.447	10	2.03	8.9
		*qBW-3*	17	43.872	60.091	2	2.39	10.4
		*qBW-4*	19	72.613	72.613	1	2.49	10.8

## Discussion

*E. carinicauda* is an economic marine shrimp species in eastern China. Given its economic importance, great researches have been made to investigate the genomic characteristics of this species. In the present study, a total of 294.46 Gb high-quality reads were obtained, covering approximately 31.57-fold genome size of *E. carinicauda*. The genome size of *E. carinicauda* was estimated to 9.33 Gb based on the K-mer analysis, which is relatively larger than the previous results and many commonly recognized decapod shrimps [30]. The genome size of *E. carinicauda* is higher than those of penaeid shrimps [20, 31], caridean shrimp *Neocaridina denticulate* [32], *Eriocheir sinensis* [33] and *Portunus trituberculatus* [34] by 4.0-, 3.1-, 8.2- and 11.3-folds, respectively. The genome survey analysis revealed the complexity of the *E. carinicauda* genome. Approximately 76.62% of the genome was occupied by repetitive sequences, which was very similar to the *L. vannamei* genome [20] and the *N. denticulate* genome [32]. A total of 31371476 contigs with N50 length of 422 bp was obtained, which was higher than that of previous study [30]. And a total of 65772 unigenes with mean length of 1386 bp were obtained, which was relatively smaller than those of decapod shrimps [30].

The reciprocal-cross backcross population of *E. carinicauda* were firstly obtained for construction of genetic linkage map and the growth traits were analyzed. Significant correlations among five growth traits (BL, BW, CL, CW and CH) have been reported in other species, such as Atlantic salmon [35] and Zhikong Scallop [36]. Similarly with our study, five growth traits were closely related to each other, with the highest correlation coefficient (r = 0.96) between BL and BW, and relatively low correlation coefficient (r = 0.81) between CW and other traits.

High-resolution genetic linkage map is an essential tool for research of genetics and genomics, such as comparative genome analysis and QTL fine mapping [37]. SNPs are especially suitable for genetic linkage map construction, which representing the most abundant and stable form of genetic variation in most genomes [38]. In this study, 301314 polymorphic SLAF tags were developed from 4 *E. carinicauda* parents and 200 offspring individuals. 4050 SNPs distributed throughout the backcross genetic map and 6334 SNPs distributed throughout the reverse backcross genetic map, providing a large number of genomic and genetic resources for *E. carinicauda*. We successfully constructed the first high-resolution genetic linkage map with 45 linkage groups, which was in accordance with karyotypes of *E. carinicauda.* The total length of the backcross map was 2939.27 cM with an average distance between adjacent markers of 0.73 cM, and the total length of the reverse backcross map was 3460.28 cM with an average distance between adjacent markers of 0.55 cM. The mapping resolution of reciprocal backcross population exceeded those of *P. monodon* (0.9 cM) [19], and similar to *L. vannamei* (0.7 cM) [20], *P. trituberculatus* (0.51 cM) [34] and *E. sinensis* (0.49 cM) [22]. Additionally, the marker developed in our study contained a 200 bp terminal sequences, which provided enough information for trait-related QTLs verfication and comparative genome analysis.

To identify potential growth-related genes, we compared the detected QTLs with the scaffold assembly from genome survey and transcriptome of *E. carinicauda* [39]. Based on the integrated map, 91 markers could be aligned to genome assemblies or transcript sequences in the backcross map, and 13 of 91 markers could annotated candidate genes via blast in public databases. Meanwhile, 156 markers could be aligned to genome assemblies or transcript sequences in the reverse backcross map, and 25 of 156 markers could annotated candidate genes via blast in public databases. These aligned genes were related to many important physiological processes and functions, such as protein kinase activity [40], regulation of apoptotic process [41] and sequence-specific DNA binding transcription factor activity [42]. These annotated genes provide candidate information for analysis of economic traits in *E. carinicauda*.

QTL mapping is a very effective strategy to locate trait-related genes for MAS in genetic breeding of aquaculture species [36]. The high-resolution genetic map constructed in this study provided important tools for performing QTL fine mapping for economical traits of *E. carinicauda*. In the present study, 7 QTLs associated with growth traits were found to be distributed on three LGs (LG1, LG2 and LG20) in the backcross linkage map. And 16 QTLs associated with growth traits were found to be distributed on six LGs (LG1, LG10, LG13, LG17, LG19, LG24 and LG38) in the reverse backcross linkage map. Interestingly, most of the QTLs were concentrated within a narrow region (cluster) on the LGs. In the backcross linkage map, three QTLs were clustered together (47.024–79.283 cM) on LG2 corresponding to assembly contig85361652 (2.2 kb) and scaffold1051673 (6.4 kb). And two QTLs were found in another cluster (0–1.826 cM) on LG20, corresponding to scaffold1658074 (1.8 kb). Meanwhile, in the reverse backcross map, three QTLs were clustered together (60.091–60.576 cM) on LG17 corresponding to assembly contig84295559 (1.3 kb), scaffold234386 (1.2 kb) and scaffold037331 (3.7 kb). Additionally, three QTLs were also clustered together (90.716–91.096 cM) on LG19, indicating that the growth traits may be controlled by the same genes. The quite small genetic and physical distances among QTLs in specific clusters suggested that the individual clusters might be highly effective QTLs. The results were in accordance with the previous results in *P. trituberculatus* [34]. The demonstration of one trait controlled by a few significant QTLs with higher PVE values was consistent with the characteristics of growth traits controlled by several major genes with higher heritability [43, 44].

## Conclusions

In conclusion, genome size of *E. carinicauda* was estimated as 9.33 Gb with remarkably low heterozygosity. Large-scale SNPs identified and genotyped via SLAF-seq technology were used to construct a high-resolution genetic map of the ridgetail white prawn. The developed genetic map is the most comprehensive genetic map to date for this species. Based on SNP mapping analysis, we identified 23 positive QTLs for growth traits that will be helpful to clarifying the genetic mechanism of growth regulation in the ridgetail white prawn. The obtained SNPs and high-resolution linkage map, coupled with genome survey and transcriptome established an important platform for QTL mapping and also provided an extremely useful resource for future molecular breeding efforts such as genome selection.

## Methods

### Genome survey analysis

DNA samples of *E. carinicauda* was extracted from the muscles of parent of backcross family for sequencing. Six paired-end DNA libraries with an insert size of 270 bp were constructed following the Illumina operating protocols. The paired-end sequencing was performed on the Illumina Hiseq 4000 platform (Illumina, Inc.; San Diego, CA, USA). The raw data were trimmed to filter out low-quality reads and adapter contaminates using NGS QC Toolkit [45]. De novo assembly was performed to obtain contigs using SOAP denovo software [46] (https://sourceforge.net/projects/soapdenovo2/files/SOAPdenovo2/) with the following parameters: the k value in K-mer was set at 23, unsolve repeats by reads fill gaps in scaffolds.

### Mapping population and phenotype data

The reciprocal-cross backcross families of *E. carinicauda* were constructed in the breeding center of Yellow Sea Fisheries Research Institute, CAFS. Firstly, A F_1_ full-sib family was created from male parent prawn from Rizhao population and female prawn from Xiangshan population. Then, cross BC_1_ family was obtained from the female F_1_ individuals and male parent, and reverse cross BC_1_ family come from the male F_1_ individuals and female parent. Finally, two reciprocal-cross backcross families were obtained after 3 months of growth. The parents prawn and BC_1_ progenies were raised in a 200 L and 500 L tanks respectively and fed four times daily under standard feeding management (5–10% feed/body weight ratio daily). The oxygen level in sea water was maintained at 5 mg/L or above. Growth-related traits including body weight (BW), body length (BL), carapace length (CL), width (CW) and height (CH) of 200 BC_1_ progenies randomly selected after 3 months of growth were measured for each individual. Statistical analysis of the growth traits data was conducted with SPSS 17.0.

DNA samples were extracted from the muscles of the parental prawns and 200 BC_1_ progenies using traditional phenol–chloroform extraction in combination with RNase treatment. Before construction of SLAF-seq libraries, all DNA samples were quantified using a NanoDrop 1000 spectrophotometer (Thermo Scientific, Wilmington, DE, USA), and the concentrations were adjusted to 50 ng/μL.

### SLAF library construction and high-throughput sequencing

Based on the size of *E. carinicauda* genome, content of GC and in-silico analysis, endonuclease *Hae* III were used to digest the genome. SLAF library were constructed as described by Sun et al. [23] with minor modifications. For the BC_1_ population, endonuclease *Hae III* (New England Biolabs, NEB, USA) were chosen to digest the genomic DNA of *E. carinicauda*. Subsequently, a single nucleotide (A) overhang was attached to the digested fragments by using Klenow Fragment (3′ → 5′ exo^−^) (NEB, USA) and dATP at 37 °C. PAGE-purified duplex tag-labelled sequencing adapters (Life Technologies, USA) were then ligated to the A-tailed fragments using T_4_ DNA ligase. PCR was performed using diluted restriction-ligation DNA samples, dNTP, Q5® High-Fidelity DNA Polymerase and PCR primers (Forward primer: 5′-AATGATACGGCGACCACCGA-3′, reverse primer: 5′-CAAGCAGAAGACGGCATACG-3′) (PAGE-purified, Life Technologies, USA). The PCR products were purified by using Agencourt AMPure XP beads (Beckman Coulter, High Wycombe, UK) and then pooled. The pooled samples were separated using 2% agarose gel electrophoresis. The fragments of 364 to 444 bp (with indexes and adaptors) in size were excised and purified using a QIAquick Gel Extraction Kit (Qiagen, Hilden, Germany). The gel-purified products were then diluted and pair-end sequenced (Each end 125 bp) on Illumina HiSeq 2500 system (Illumina, Inc.; San Diego, CA, USA) according to the manufacturer’s protocols. To avoid false positive reads, the sequence error rate was estimated using the data of *Oryza sativa* as a control. The ratio of high quality reads with quality scores greater than Q30 (indicating a 1% chance of an error, and thus 99% confidence) in the raw reads and GC amounts were calculated for quality control.

### SNP calling and genotyping

After filtering out the low-quality reads (quality score < 30e), the remaining reads were sorted to each progeny according to duplex barcode sequences. Then each of the high-quality read was trimmed off 5-bp terminal position, sequences clustered by similarity above 90% were defined as one SLAF locus [47]. Genome Analysis Toolkit (GATK) [48] and Sequence Alignment/Map tools (SAMtools) [43] was used for calling the SNPs. Local realignment was performed to avoid false alignments near InDels. The “Unified Genotyper” module of GATK was used for variant calling. Both SAMtools and GATK tools were used to identify SNPs, and their intersection was merged as the candidate SNP dataset. Only biallelic SNPs were identified as the final SNP dataset. Polymorphic SNP markers were converted to four segregation patterns (hk × hk, lm × ll, nn × np and aa × bb). The reciprocal-cross backcross families of *E. carinicauda* were obtained from the crosses between parents and F_1_ individuals with genotype aa or bb. Thus, only the SNPs whose segregation patterns were aa × bb were used to construct the genetic linkage map. The average sequencing depths of SNPs were more than 18-fold in the parents and greater than 6-fold in the progeny, respectively. And a progeny contained more than 70% of the SNPs in both parents, i.e., 70% integrity of SNPs in individuals. A χ^2^ test was implemented for each SNP with a null hypothesis that the two alleles at a locus segregated with 1:1 ratio in our BC population. All SNPs that significantly deviated from this ratio (*P* < 0.001) were eliminited from the SNP dataset.

### Genetic linkage map construction

To ensure the quality of the genetic map, HighMap strategy was utilized to sort the SNP markers and correct genotyping errors within LGs [44]. Marker loci were distributed primarily into LGs by the modified logarithm of odds (MLOD) scores > 5.0 and a maximum recombination fraction of 0.4. MSTmap algorithms [49] and SMOOTH algorithms [50] were chosen to order the SNP markers and correct genotyping errors, respectively. The LGs were constructed as follows: Primary marker orders were first obtained by their location on the chromosomes according to the relationship of the ordered markers, and genotyping errors or deletions were corrected using the SMOOTH algorithm; then, MSTmap was used to sort the map and SMOOTH was reused to correct the newly ordered genotypes. The processes were conducted repetitively to ensure the accuracy of marker order and map distances, finally high-quality maps were obtained after four or more processes.

### Integration of genetic linkage map, genome and transcriptome

The reference transcriptome unigenes were de novo assembled using the obtained reads from our previous work [39]. The clean reads were assembled with Trinity program as reported previously [51], followed by TIGR Gene Indices Clustering Tools (TGICL) [52]. The reads were then mapped back to contigs with paired-end reads to detect contigs from the same transcript and the distances between these contigs. The letter N was used to connect each two contigs to represent unknown sequences, and then for Scaffold. Finally, sequences were obtained that lacked N and could not be extended on either end [53], and were defined as unigenes. These unigenes were aligned by BlastX to public databases including the NCBI, the Swiss-Prot database, the KEGG database, and the COG database (E-value ≤1.0 × 10^− 5^).

An integrated map was constructed using the map markers, transcriptome unigenes and genomic scaffolds/contigs mainly via BLAT and the Circos tool [54]. Before BLAT analyzing, the Repeat-Masker software was run to increase the accuracy of alignment by masking homologous repeats [55]. Three sets of data were integrated and aligned to each other by BLAT analysis. The outer ring of the integration map represented the physical distance of each LG, which was the total length of genomic scaffold/contig alignment to the LG with the default parameters (minMatch 2, minScore 30, minIdentity 90, maxGap 2). The middle ring represented the alignment relation between SNP markers and genomic scaffold/contig. If the two ends of a marker matched different scaffold/contig sequences, then the marker was linked to the two scaffold/contig sequences. The inner ring represented the alignment relation between transcriptome unigenes and genome scaffold/contig sequences, and calculated the number of unigenes per kb of genome sequence.

### QTL analysis for growth-related traits

The growth traits phenotypic data of backcross population were shown in Additional file 1: Table S1. QTL mapping analysis were conducted using MapQTL 4.0 software by the composite interval mapping (CIM) method [27, 56]. CIM method was used with a walking speed of 1 cM. Two-LOD support intervals were constructed with 95% confidence intervals [57]. The significance of each QTL interval was verified using likelihood-ratio statistic (LOD). The threshold of the LOD score for significance (*P* = 0.05) was detected using 1000 permutations. Calculation of the percentage of growth-traits variation explained (*PVE*) by each QTL (Expl. %) was conducted with MapQTL 4.0 based on the variation within the mapping population.

## Additional files

Additional file 1: **Table S1.** Phenotypic data of sequenced individuals from BC_1_ and reverse BC_1_ families of *E. carinicauda*. (XLSX 24 kb)

Additional file 2: **Table S2.** The predicted genes from the genome survey of *E. carinicauda*. (XLSX 9232 kb)

Additional file 3: **Table S3.** The SNP marker number and the read depth of each genotyped marker in the backcross mapping family. (XLSX 15 kb)

Additional file 4: **Table S4.** The SNP marker number and the read depth of each genotyped marker in the reverse backcross mapping family. (XLSX 15 kb)

Additional file 5: **Table S5.** Marker information of Backcross linkage map. (XLSX 107 kb)

Additional file 6: **Table S6.** Marker information of reverse Backcross linkage map. (XLSX 151 kb)

Additional file 7: **Table S7.** Markers of Backcross linkage map anchored to scaffolds. (XLSX 151 kb)

Additional file 8: **Table S8.** Markers of Backcross linkage map anchored to transcriptome. (XLSX 28 kb)

Additional file 9: **Table S9.** Markers of reverse Backcross linkage map anchored to scaffolds. (XLSX 227 kb)

Additional file 10: **Table S10.** Markers of reverse Backcross linkage map anchored to transcriptome. (XLSX 39 kb)

## Data Availability

The datasets supporting the findings of this article are included within the article and its supplementary information files. The raw sequence data from this study were deposited at the NCBI Nucleotide database with the accession Number QUOF000000000.1.

## Abbreviations

BC: Backcross
LGs: Linkage Groups
LOD: Logarithm of Odds
MAS: Marker-Assisted Selection
NGS: Next Generation Sequencing
PVE: Phenotypic Variance Explained
QTL: Quantitative Trait Loci
SLAF: Specific-Locus Amplified Fragment sequencing
SNP: Single Nucleotide Polymorphism
